# Transient Ureteral Obstruction Prevents against Kidney Ischemia/Reperfusion Injury via Hypoxia-Inducible Factor (HIF)-2α Activation

**DOI:** 10.1371/journal.pone.0029876

**Published:** 2012-01-25

**Authors:** Shun Zhang, Cong-Hui Han, Xiao-Song Chen, Ming Zhang, Long-Mei Xu, Jian-Jun Zhang, Qiang Xia

**Affiliations:** 1 Department of Transplantation and Hepatic Surgery, Renji Hospital, Shanghai Jiaotong University School of Medicine, Shanghai, People's Republic of China; 2 Department of Urology, Xuzhou Central Hospital, Xuzhou Medical University School of Clinical Medicine, Xuzhou, People's Republic of China; National Cancer Institute, United States of America

## Abstract

Although the protective effect of transient ureteral obstruction (UO) prior to ischemia on subsequent renal ischemia/reperfusion (I/R) injury has been documented, the underlying molecular mechanism remains to be understood. We showed in the current study that 24 h of UO led to renal tubular hypoxia in the ipsilateral kidney in mice, with the accumulation of hypoxia-inducible factor (HIF)-2α, which lasted for a week after the release of UO. To address the functions of HIF-2α in UO-mediated protection of renal IRI, we utilized the Mx-Cre/loxP recombination system to knock out target genes. Inactivation of HIF-2α, but not HIF-1α blunted the renal protective effects of UO, as demonstrated by much higher serum creatinine level and severer histological damage. UO failed to prevent postischemic neutrophil infiltration and apoptosis induction in HIF-2α knockout mice, which also diminished the postobstructive up-regulation of the protective molecule, heat shock protein (HSP)-27. The renal protective effects of UO were associated with the improvement of the postischemic recovery of intra-renal microvascular blood flow, which was also dependent on the activation of HIF-2α. Our results demonstrated that UO protected the kidney via activation of HIF-2α, which reduced tubular damages via preservation of adequate renal microvascular perfusion after ischemia. Thus, preconditional HIF-2α activation might serve as a novel therapeutic strategy for the treatment of ischemic acute renal failure.

## Introduction

Ischemia and reperfusion injury (IRI) is unavoidable in renal transplantation, and often causes acute renal failure, which is associated with prolonged hospitalization and high mortality [1], [2]. Novel effective interventional strategies not only help to improve the clinical outcome, but also promote understanding of renal pathophysiology.

It was reported [3] that transient ureteral obstruction (UO) protected the kidney against the subsequent exposure to ischemia and reperfusion (I/R) insults. This protection was not related to uremia or humoral factors, since unilateral UO conferred protection on the ipsilateral kidney, but not the contralateral one [4]. The authors also reported that prior UO resulted in reduced postischemic outer medullary congestion and leukocyte infiltration. These results were very similar to those with preconditional activation of hypoxia-inducible factors (HIFs) [5], [6].

HIFs are master regulators of oxygen homeostasis and stimulate numerous genes important for energy metabolism, glucose transport, vasomotor regulation, angiogenic growth and erythropoiesis, after activation [7], [8]. HIFs are heterodimeric factors consisting of a constitutive β subunit, HIF-1β and one of three alternative α subunits, HIF-1α, HIF-2α, and HIF-3α [9], [10]. Under normoxic conditions, HIF-α subunits have a very short half-life. Cells continuously synthesize but rapidly degrade HIF-α protein. Under conditions of hypoxia, HIF-α is no longer degraded, and translocates into the nucleus, where it dimerizes with HIF-1β to form the active HIF complex. HIF activates the transcription of target genes by binding to hypoxia-response elements (HREs) in their promoter or enhancer regions [11]. According to recent reports [12], the most widely expressed and best characterized α subunit in the hypoxic/ischemic kidney is HIF-1α, and its expression has been described in both tubular and glomerular epithelial cells. HIF-2α is also induced in the hypoxic kidney, but is localized in glomerular cells, endothelial cells, and fibroblasts [13], [14]. Importantly, HIF-1α and HIF-2α appear to regulate distinct subsets of target genes [15].

It has been reported [16] that UO kidneys are hypoxic and display protein and gene expression changes consistent with HIF activation, the role of which in the protection of renal IRI has not been clearly defined so far. In this study, we inactivated HIF-1α and HIF-2α genes by the Cre-loxP conditional gene disruption system to create HIF-1α or HIF-2α knockout mice, as well as HIF-1α/2α double knockout (DKO) mice, and subjected them to transient UO followed by renal I/R, to explore the role of HIFs in the renoprotective effects of UO.

## Materials and Methods

### Ethic statements

All animal experiments have been conducted according to standard use protocols, animal welfare regulations and the institutional guidelines of Shanghai Jiaotong University School of Medicine and the Regulations for Practice of Experimental Animals (issued by Scientific and Technical Committee, P.R.China, 1988). All the procedures described were approved by the Animal Use and Care Committee of Shanghai Jiaotong University School of Medicine (approval number: SYKX-2008-0050). All surgery was performed under sodium pentobarbital anesthesia. Analgesia used was bupivacaine(0.5%), a long acting local analgesic, immediately after surgery and only once. Several drops of bupivacaine were dripped on the suture line after the muscle layer was closed, and before the closure of skin wound. All these efforts were made to minimize suffering.

### Mice

The Cre/loxP recombination system was used to generate HIF-1α^−/−^, HIF-2α^−/−^, or HIF-1α/2α double knockout mice. Mice containing loxP-flanked HIF-1α exon 2 (HIF-1α^loxP/loxP^, stock number: 007561), loxP-flanked HIF-2α exon 2 (HIF-2α^loxP/loxP^, stock number: 008407) and Mx promoter sequence-modified Cre recombinase gene (Mx-cre, stock number: 003556) were from the Jackson Laboratory (Bar Harbor, Maine USA). After a mating of HIF-1α^loxP/loxP^ and Mx-cre strains and a second mating of their progeny, mice that were homozygous for the HIF-1α floxed allele and also carried the Mx-cre transgene were generated (Mx^+^HIF-1α^loxP/loxP^). Mx^+^HIF-1α^loxP/loxP^ mice were then backcrossed to the Mx^−^HIF-1α^loxP/loxP^ mice to generate both Mx^+^ mice (deletable) and Mx^−^ littermates (nondeletable). To mutate the target gene, 8-week old mice were administered intraperitoneal injections of 400-µg poly deoxyinosinic/deoxycytidylic acid (pIpC) every 4 days for a total of three injections. Mx^+^HIF-1α^loxP/loxP^ mice that had received injections of pIpC were hereafter referred to as HIF-1α^−/−^ mice. The generation of HIF-2α^−/−^ mice and the matching controls was as described above. Mx^−^HIF-1α^loxP/loxP^ and Mx^−^HIF-2α^loxP/loxP^ mice that had received injections of pIpC served as controls in all the following experiments and were referred to as wild-type (WT) mice.

Mx^+^HIF-1α^loxP/loxP^ mice were then mated with Mx^+^HIF-2α^loxP/loxP^ mice. After a second mating of their progeny, mice that were homozygous for both HIF-1α and HIF-2α floxed allele and also carried the Mx-cre transgene were selected by genotyping. Mx^+^HIF-1α^loxP/loxP^ HIF-2α^loxP/loxP^ mice that had received injections of pIpC were deficient in both HIF-1α and HIF-2α genes, hereafter were referred to as double knockout (DKO) mice.

Male mice, 8–14 weeks of age and weighing 20–28 g, were used in the present study.

### Genotyping

Genomic DNA was isolated from tail biopsies. Genotyping was performed using PCR which had been described previously. The HIF-1α^loxP/loxP^ and the wild-type alleles were detected using the following primers: 5′- GGAGCTATCTCTCTAGACC -3′ and 5′- GCAGTTAAGAGCACTAGTTG -3′, which generated a 250 bp product in floxed allele and 215 bp product in wild type [17]. To distinguish HIF-2α^loxP/loxP^ mice from wild type by multiplex PCR, the primers were as follows: P1: 5′-CAGGCAGTATGCCTGGCTAATTCCAGTT-3′; P2: 5′-CTTCTTCCATCATCTGGGATCTGGGACT-3′; P3: 5′-GCTAACACTGTACTGTCTGAAAGAGTAGC-3′. The wild type allele produced a 410-bp fragment (P1 and P2), the 2-loxP allele produced a 444-bp fragment (P1 and P2), and the 1-loxP allele produced a 340-bp fragment (P1 and P3) [18]. The Mx-cre transgene was detected using the primers 5′-ACCTGAAGATGTTCGCGATTATCT-3′, and 5′-ACCGTCAGTACGTGAGATATCTT-3′, which amplified a 370-bp fragment [19].

### Transient unilateral ureteral obstruction (UO)

A transient UO procedure was used as described [3], with modifications. After anesthesia a midline laparotomy was made. The left ureter was identified and surrounded by a 7-0 silk suture 1 cm below its renal origin, and then was ligated by tying a one-loop shoelace knot. The right ureter was left undisturbed. The suture end, which was left outside the abdominal wall after the incision was closed, would be slipped and removed 24 h after the operation to release the ureter from obstruction. 0 (immediately), 2, 4 or 7 days after release of obstruction, these mice were subjected to renal I/R insults. Sham controls underwent the same surgical procedures but without tying the knot, hereafter were referred to as non-UO controls.

### Renal ischemia-reperfusion model (IR)

Anesthesia was induced with sodium pentobarbital (60 mg/kg body weight i.p.). Mice were placed on a temperature-controlled heating table with a rectal thermometer probe attached to a thermal feedback controller (ALC-HTP Homeothermic System, Shanghai Alcott Biotech Co. Ltd, China) to maintain rectal temperature at 36°C. A warm renal IR model was used as described [20], with minor modifications. In brief, following a midline abdominal incision, right nephrectomy was performed. After intraperitoneal injection of heparin (50 U/kg), left renal pedicle was localized and clamped for 25 min using an atraumatic micro-vascular clamp. After inspection for signs of ischemia, animals were covered with surgical dressing to keep stable intraperitoneal temperature. After removal of the clamp, restoration of blood flow was inspected visually. Sham controls underwent same surgical procedures but without vascular occlusion, hereafter were referred to as non-IR controls. Animals were killed 6 h or 24 h after reperfusion by exsanguination, to obtain blood and renal samples for further analyses.

### Tissue oxygen partial pressure (tPO_2_) measurements and laser doppler flowmetry (LDF) monitoring in kidneys

A large-area-surface O_2_ probe, which was connected to a tissue oxygen monitoring system (OxyLab pO_2_ system, Oxford Optronics, UK), was used for tPO_2_ monitoring in mouse kidneys, as described previously [21], [22]. Measurements were performed in both kidneys 24 h after left UO, without release of obstruction. The inferior pole of the kidney was punctured using a 22-gauge needle and the O_2_ probe was inserted to a depth of 3 mm. Since the O_2_ probe measured oxygenation along the shaft of the catheter, i.e., away from the tip, it was actually sampling from a depth of 2 mm. We established in preliminary experiments that such an insertion depth enabled measurement of tPO_2_ within renal outer medulla. The measurement began 30 minutes after the probe was inserted and continued for 10 minutes. The values of ten minutes collected by the probe were expressed as a mean pO_2_ value (mmHg) over the period of observation. The core temperature was maintained at 36°C throughout the procedures. It's worth notice that core body temperature control is very important to the measurement. The oxygen tension levels vary a lot, and tend to be lower without maintaining the normal body temperature.

For renal tissue blood perfusion monitoring, a pO_2_/Flow Bare-Fibre sensor, which was connected to an OxyLab LDF instrument (Oxford Optronix, UK), was inserted into renal outer medulla to enable monitoring of continuous microvascular blood flow, as described previously [22]. The measurement began 30 minutes after the probe was inserted and continued for 10 minutes with the core temperature maintained at 36°C. The values of ten minutes collected by the probe were expressed as a mean value of blood perfusion units (BPU) over the period of observation. Baseline renal blood flow was obtained by monitoring the microvascular blood flow in the right kidney before UO or sham procedures. After the initiation of reperfusion, the blood flow in the left kidney was measured at the indicated time points, and the mean value versus the baseline result was defined as relative renal perfusion.

### Biochemical analyses

Arterial blood was collected by direct puncture of arteriae aorta. Serum creatinine (Cr) was measured with a standard clinical automatic analyzer (Siemens Dade behring dimension xpand).

### Histology and histomorphological scoring of acute tubular injury

Kidney tissues were fixed in 10% neutral buffered formalin overnight, dehydrated, embedded in paraffin and sectioned at 5 µm. For histological analysis, sections were stained with Periodic Acid-Schiff (PAS). Samples were analyzed for tubular cell necrosis, tubular dilation, intratubular cell detachment, and cast formation (original magnification ×200) and were all evaluated in a blinded manner by a nephropathologist. Abnormalities were graded by a semiquantitative histomorphological scoring system from 0 to 4, as described previously [23]. At least 3 fields per section were evaluated.

### Polymorphonuclear leukocyte infiltration (MPO activity)

Renal sections were processed for immunohistochemical localization of myeloperoxidase (MPO, polyclonal rabbit antibody; Novus Biologicals, NBP1-42591), and were then visualized with diaminobenzadine (DAB) and counterstained with hematoxylin. Polymorphonuclear leukocyte (PMN) infiltration was scored semiquantitatively on a scale of 1 (none) to 4 (severe), as described previously [24].

### Terminal deoxynucleotidyl transferase-mediated 2′-deoxyuridine 5′-triphosphate nick-end labeling assay (TUNEL)

Apoptotic cells in formalin-fixed, paraffin-embedded kidney tissue sections were identified with ApopTag™ Fluorescein In Situ Apoptosis Detection Kit S7110 (Chemicon International), according to the manufacturer's protocol. Cells with nuclear positive staining by fluorescent antibodies for DNA fragmentation were visualized directly by a fluorescence microscopy and counted (original magnification ×400). At least 3 fields per section were examined.

### Quantitative western blot analysis

Western blot analysis of HIF-1α and HIF-2α was performed as described previously [25]. Nuclear extracts were isolated from harvested whole kidneys using NE-PER Nuclear and Cytoplasmic Extraction Reagents (Product Number 78833, Pierce Biotechnology, Inc., USA.), supplemented with Complete Protease Inhibitor Cocktail Tablets (Roche, Indianapolis, IN). Nuclear protein fractions were electrophoresed on 10% SDS-PAGE under reducing conditions and transferred to a nitrocellulose membrane (Whatman) by standard procedures. Membranes were blocked with LI-COR blocking buffer (LI-COR Biosciences, Lincoln, NE). Membranes were then incubated with the same blocking solution containing rabbit polyclonal primary antibodies against HIF-1α (1∶500, NB100-479, Novus Biologicals) and HIF-2α (1∶500, ab199, Abcam). After washing, membranes were incubated at room temperature for 1 h in TBS/0.05% Tween 20 buffer with the IRDye800 secondary antibodies (1∶10000; LI-COR Biosciences) and then washed again in TBS/0.05% Tween 20 for 3 times. The blot was visualized using an Odyssey infrared imaging system (LI-COR Biosciences). Samples were corrected for background and quantified using Odyssey software. All values were normalized to a loading control TATA binding protein (TBP, 1∶2000, ab818, Abcam) and expressed as fold increase relative to control.

For heat shock protein (HSP)-27 measurements, harvested kidneys were homogenized and lysed with cell lysis buffer, which contained 1 protease inhibitor cocktail tablet per 10 mL of Lysis Reagents (Complete; Roche, Indianapolis, IN). Solutions were then clarified by centrifugation (25 minutes at 16,000 g). Solubilized proteins were then resolved on a 10% SDS-polyacrylamide gel and transferred to nitrocellulose membranes (Whatman). After blocked with LI-COR blocking buffer, blots were incubated with anti-HSP-27 (1∶500, #2442, Cell Signaling Technology, Inc.) and anti-β-actin (1∶2000, Santa cruz biotechnology, inc.) antibodies. After incubation with secondary antibodies, blots were developed as described above. HSP-27 expression levels were normalized to β-actin expression levels.

### Statistics

All values were reported as the mean ± standard deviation (SD). Data were analyzed with a one-way ANOVA with subsequent Student-Newman-Keul's test or Student's t-test where applicable. Statistical significance was set at P<0.05.

## Results

### UO led to renal tubular hypoxia

Since UO causes blockage of urine flow from the kidney and thus induces a high pressure state within the intrarenal microstructures, we hypothesized that UO might change the balance between local oxygen supply and consumption, and lead to hypoxia. To confirm this, we employed OxyLab pO_2_ system, which had a proven record of success in a variety of applications [26]–[30], to evaluate the renal tissue oxygenation after UO.

**Figure 1:**
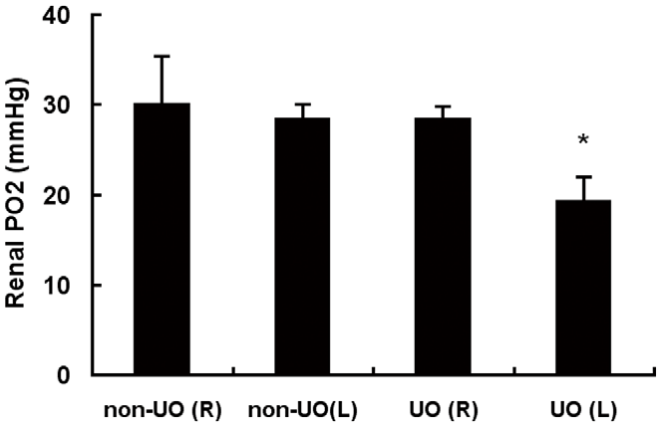
Effect of 24 h UO on renal tissue oxygen partial pressure (tPO_2_) level. WT mice were subjected to either non-UO sham surgery or left UO. After 24 hours, bilateral renal tPO_2_ measurements were performed without release of UO. There were 5 mice in each group. Values were expressed as mean ± SD. *, p<0.05 versus the other three groups. L, left kidney; R, right kidney.

The left and right kidneys in non-UO control mice manifested similar tissue oxygen tensions in outer medulla 24 h after the sham operation, which were comparable with the baseline levels derived from those without prior operations (data not shown). Left UO didn't change the tPO_2_ level in the right kidney, but led to a fall from 28.54±1.47 to 19.35±2.67 mmHg of the oxygen tension in the ipsilateral kidney 24 hours later (Figure 1).

### 24 hours of UO caused an accumulation of HIF-2α, but not HIF-1α

To determine whether the hypoxic state induced by UO could lead to accumulation of HIF-1α and HIF-2α, and how long this accumulation could persist after release of obstruction, we evaluated renal HIF-1α and HIF-2α protein levels by quantitative western blot analyses on day 0 (immediately), day 2, day 4 and day 7 after release of obstruction.

**Figure 2:**
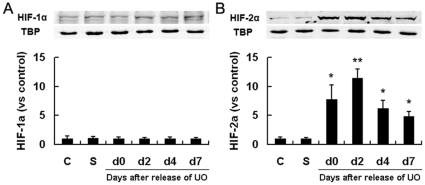
Effect of 24 h UO on renal HIF-1α/2α expression. WT mice were exposed to either sham-operation or left UO, which continued for 24 hours, and then was released. The left kidneys were harvested immediately (d0), 2, 4 or 7 days after release of obstruction (n = 4 at each time point). Immunoblot analyses of HIF-1α and HIF-2α in left kidneys were then performed and co-detection of TBP was performed to assess equal loading. HIF protein bands were quantified and normalized to TBP. Data were expressed as mean ± SD, and the mean value obtained from non-operated control mice was arbitrarily defined as 1. *, p<0.05 versus sham-operated controls; **, p<0.05 versus all the other groups. C, non-operated controls; S, sham-operated controls.

Kidneys from non-operated controls or non-UO sham-operated mice had barely detectable levels of HIF-1α and HIF-2α, whereas left kidneys from UO mice had much higher HIF-2α levels on day 0 (24 h after initiation of obstruction), which were even further up-regulated on day 2, and then decreased on day 4. One week after release of UO, HIF-2α was still maintained at a relatively high level (Figure 2). HIF-1α levels remained unchanged, similar to those of non-UO controls at all tested time points. These results suggested that renal hypoxia resulted from UO selectively up-regulated HIF-2α and the biological effects of UO might be mediated by HIF-2α, rather than HIF-1α.

### Inactivation of HIF-2α, rather than HIF-1α, neutralized the renoprotective effects of UO

**Figure 3:**
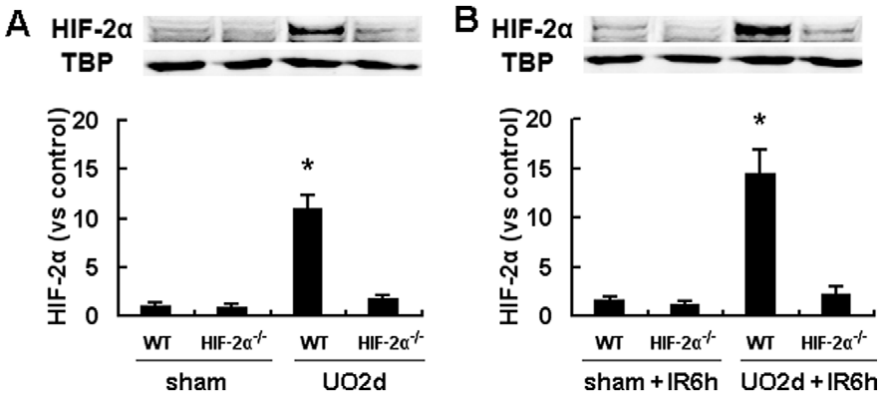
HIF-2α levels in HIF-2α^−/−^ and WT mouse kidneys. A, HIF-2α^−/−^ mice or their wild-type littermates were exposed to left UO, which continued for 24 hours, and then was released. 2 days after release of UO or at the corresponding time point in the non-UO sham-operated mice, the left kidneys were harvested and subjected to immunoblot analyses of HIF-2α and co-detection of TBP as a loading control. B, Mice were also exposed to I/R procedures before the harvest of the left kidneys to evaluate the HIF-2α levels 6 hours after the initiation of reperfusion. HIF-2α protein bands were quantified and normalized to TBP. There were 4 mice in each group and data were expressed as mean ± SD. The mean value obtained from sham-operated WT mice was arbitrarily defined as 1. *, p<0.05 versus all the other 3 groups.

**Figure 4:**
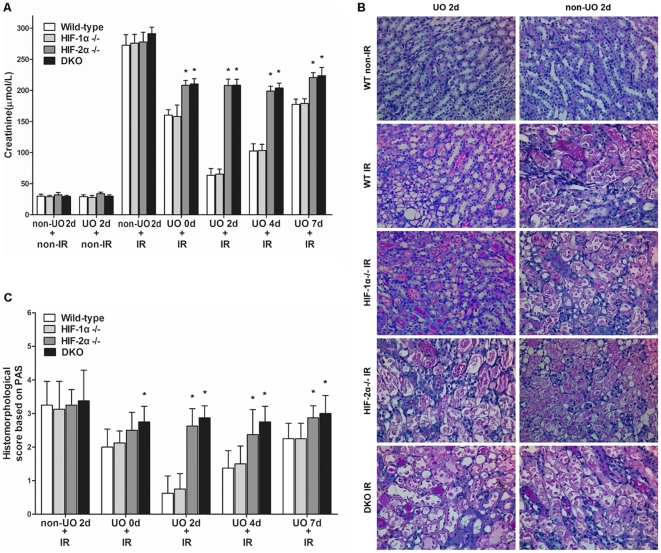
UO-mediated renoprotective effects were negated by HIF-2α knockout. Mice were exposed to either non-UO sham-operation or left UO, which continued for 24 hours, and then was released. At different time points (0 d, 2 d, 4 d, 7 d) after release of UO, animals were subjected to right nephrectomy, followed by IR (25 min of left renal ischemia and 24 h of reperfusion) or non-IR sham operation. A, concentrations of serum creatinine 24 h after the initiation of reperfusion. B, representative renal PAS-stained sections from mice of UO 2 d group and their non-UO controls (original magnification ×200). Sections from HIF-1α^−/−^, HIF-2α^−/−^ and DKO mice in non-IR groups had no positive manifestations (similar to those of WT non-IR mice) and were not shown. C, abnormalities based on PAS-stained sections was graded by a semiquantitative histomorphological scoring system from 0 to 4. All mice in non-IR groups had no tubular damage (histomorphological score: 0, data not shown). Data were expressed as mean ± SD from 6–8 animals per genotype. *, p<0.05 versus WT mice that were treated with the same procedures.

Inactivation of target genes was confirmed by immunoblot analyses. The accumulation of HIF-2α in kidney after UO or UO plus I/R procedures was largely diminished by the Mx-Cre/loxP recombination system (Figure 3). To confirm whether UO provided protection against IRI after release of obstruction in WT mice, as described previously [3], renal pedicle of the previously obstructed kidney was clamped for 25 min after right nephrectomy, and then was allowed for 24 h of reperfusion. Compared with non-UO controls, UO significantly improved renal function, as indicated by much lower serum Cr concentrations (Figure 4A). Ischemia imposed at all tested time points after release of UO resulted in significant decrease in Cr levels, but the profoundest decrease was seen on day 2. This was in line with the HIF-2α induction pattern after UO. To investigate the role of HIFs in the protective effects of UO, HIF-1α^−/−^, HIF-2α^−/−^ and DKO mice were subjected to the same procedures as described above. Although all these mice manifested similar Cr levels to WT mice without UO procedures, UO preconditioning led to discrepancies among different strains. HIF-1α^−/−^ mice also benefited from UO as WT mice did, but HIF-2α^−/−^ and DKO mice were much more susceptible to IR injury after UO.

The result of Cr levels was reinforced by histological observations. Tubular damage was assessed by PAS staining. Both WT and HIF-1α^−/−^ mice that were subjected to UO plus IR manifested much mitigated tubular injury, compared with non-UO plus IR renal sections. In contrast, both HIF-2α^−/−^ and DKO mice had much severer renal damage, even though they were also pretreated with UO (Figure 4B). Histological abnormalities of renal sections from all tested time points after UO were scored and shown in Figure 4C. UO preconditioning significantly decreased histological scores in WT and HIF-1α^−/−^ sections, whereas the effects of UO were neutralized to a great extent by HIF-2α or double knockout. Histomorphological scores of tubular damage correlated well with the serum Cr results.

These results demonstrated that renal resistance against ischemia conferred by UO was dependent on the timing of ischemia relative to release of UO, which was in line with the accumulation of HIF-2α. And HIF-2α, rather than HIF-1α knockout neutralized most protective effects of UO. Thus the renoprotective effects of UO should be attributed to the transcriptional responses induced by HIF-2α, rather than HIF-1α.

### The role of HIFs in UO-mediated reduction of postischemic neutrophil infiltration

**Figure 5:**
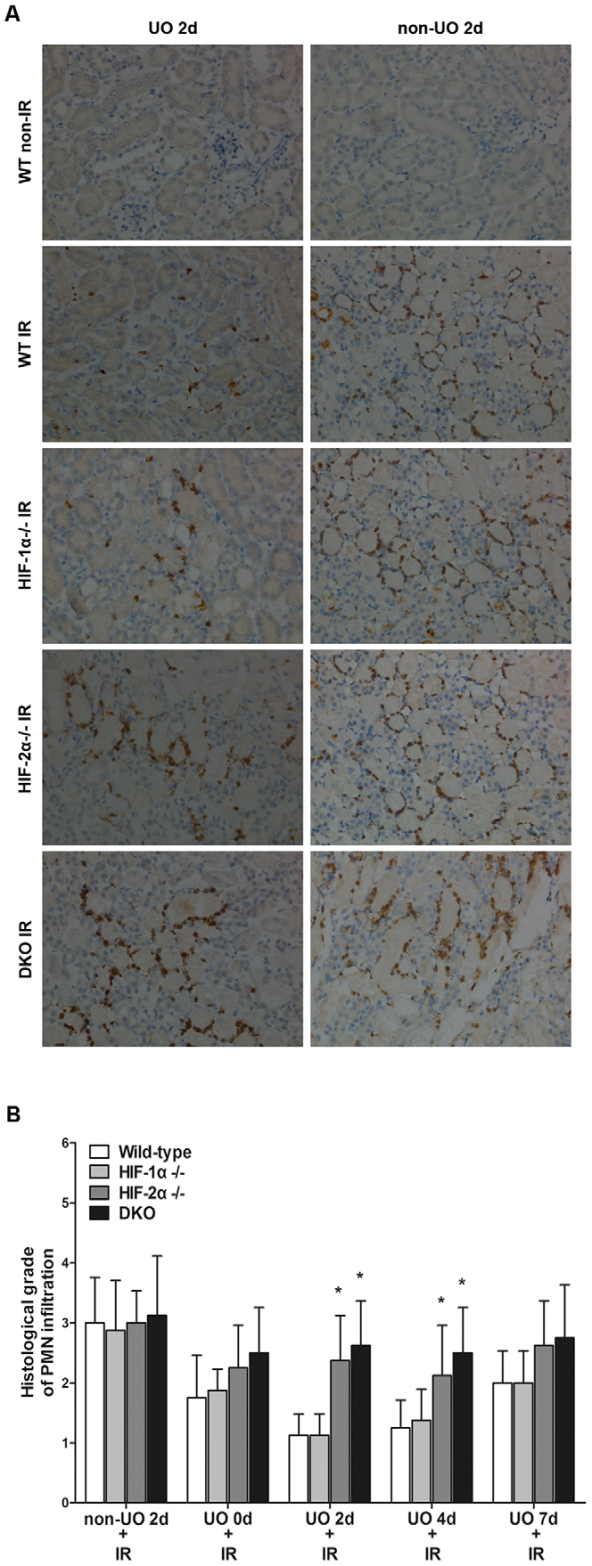
Effect of UO on post-ischemic polymorphonuclear leukocyte (PMN) infiltration in different strains. At different time points (0 d, 2 d, 4 d, 7 d) after release of UO, animals were subjected to renal IR, and then the left kidneys were harvested and immunostained for MPO activity. A, representative renal sections from mice of UO 2 d group and their non-UO controls (original magnification ×200). Sections from HIF-1α^−/−^, HIF-2α^−/−^ and DKO mice in non-IR groups had no PMN infiltration and were not shown. B, PMN infiltration was scored on a scale of 1–4. The results of different strains at all tested time points were presented. All mice that were subjected to non-IR sham operation had no PMN infiltration and the results were not shown. Data were expressed as mean ± SD from 6–8 animals per genotype. *, p<0.05 versus WT mice that were treated with the same procedures.

IR results in neutrophil recruitment, and neutrophil-mediated renal injury is an important component of renal IR injury [31]. Previous report [3] has indicated that the protective effects of UO were related to a reduction of the postischemic tissue MPO activity. To clarify the role of HIFs in this process, renal sections from different strains were also immunostained for MPO activity. Figure 5 showed that prior UO prevented most of the postischemic increase in MPO activity in WT mice, as well as in HIF-1α^−/−^ mice. This dissociated HIF-1α from UO mediated reduction of neutrophil infiltration. However, HIF-2α or double knockout negated the effects of UO on MPO activity, indicating that HIF-2α played a big part in reducing neutrophil infiltration.

### The role of HIFs in UO-mediated prevention of apoptosis

**Figure 6:**
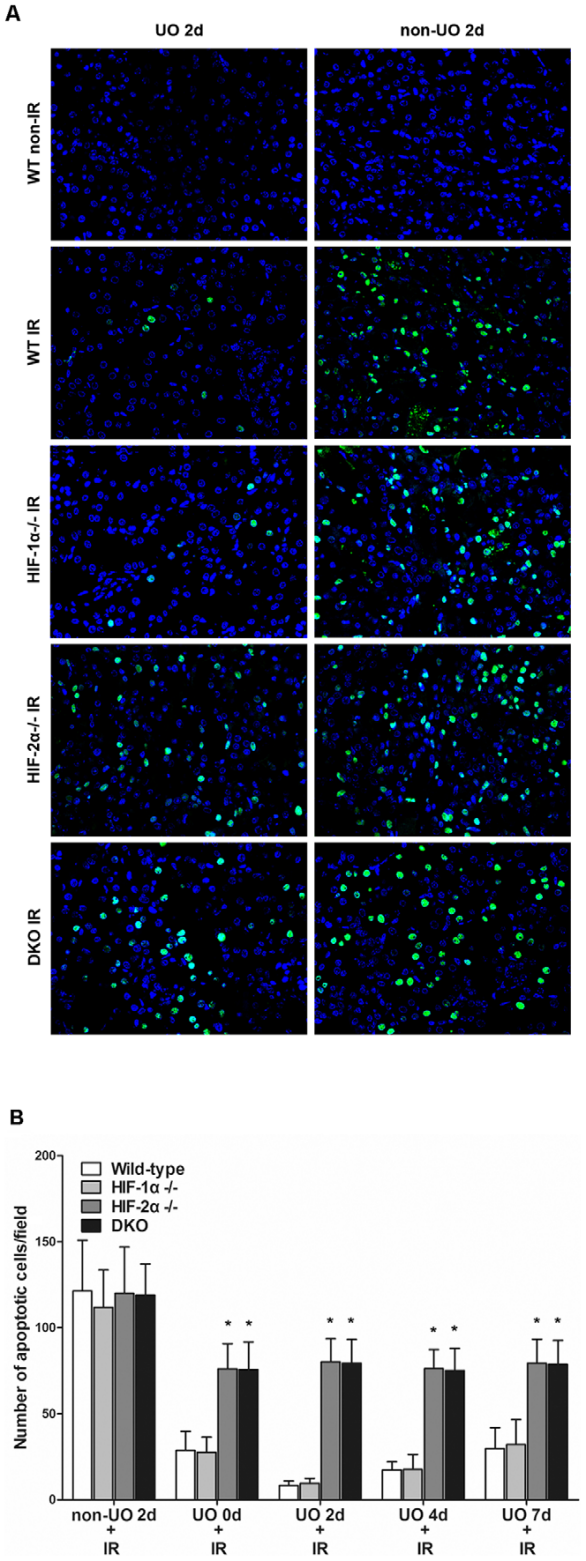
Effect of UO on apoptosis induction in different strains. A, TUNEL assay showed positive nuclear staining by fluorescent antibodies for DNA fragmentation in apoptotic cells in representative renal sections from mice of UO 2 d group and their non-UO controls (original magnification ×400). Sections from sham-operated (non-IR) HIF-1α^−/−^, HIF-2α^−/−^ and DKO mice manifested no positive staining (data not shown). B, a summary of the quantitative analysis of apoptotic cells per field. All mice that were subjected to non-IR sham operation had no apoptotic cells (data not shown). Data were expressed as mean ± SD from 6–8 animals per genotype. *, p<0.05 versus WT mice that were treated with the same procedures.

To evaluate the role of HIFs in apoptosis induction in the setting of UO-mediated renoprotection, TUNEL assay was used to detect apoptotic cells in renal sections from different strains of mice. Renal IR without UO induced similar degrees of apoptosis in all the strains, showing that without pretreatment, neither HIF-1α nor HIF-2α participated in apoptosis induction or prevention. Apoptotic cells were reduced to a great extent by UO preconditioning in WT and HIF-1α^−/−^ mice. However, UO didn't manifest such an obvious effect in HIF-2α^−/−^ or double knockout mice (Figure 6A). The degree of renal tubular apoptosis was also quantified and graphically presented in Figure 6B, to show the overall effects of UO on apoptosis prevention at all tested time points.

### Postobstructive expression of HSP-27 was compromised by HIF-2α knockout

**Figure 7:**
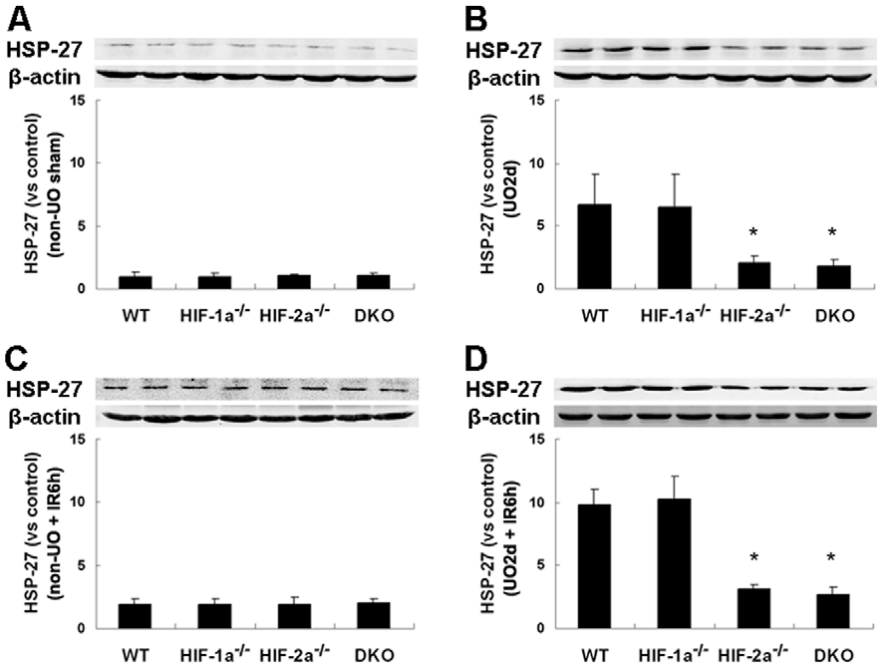
Effect of HIF-1α/2α inactivation on postobstructive and postischemic expression of HSP-27. Western blot analysis of HSP-27 in left kidneys of mice subjected to non-UO sham operation (A) or 24 h UO followed by recanalization (2 days) (B). Additional mice were subjected to non-UO procedures, followed by 25 min of left renal ischemia and 6 h of reperfusion (C), or UO 2 d, followed by the same IR procedures (D). Codetection of β-actin was performed to assess equal loading. Protein bands were quantified, and the relative density of protein bands obtained from sham-operated WT mice was arbitrarily defined as 1. Graph showed data acquired from 4 independent experiments for each mouse strain. *, p<0.05 compared with WT mice.

The up-regulated expression of HSP-27 by UO, and the protective effects of HSP-27 on renal IRI have been described [3]. However, the regulatory mechanism of HSP-27 overexpression in this setting has not been established so far. To gain insight into the relationship between HIFs and HSP-27, we analyzed renal expression of HSP-27 in wild-type and the genetically engineered mice 2 days after release of UO, as well as 6 hours after ischemic insult. As compared with non-UO controls (Figure 7A), UO led to markedly up-regulated HSP-27 levels in WT and HIF-1α^−/−^ mice, but failed to induce the same expression enhancement in HIF-2α^−/−^ and DKO mice (Figure 7B). Moreover, while IRI without UO failed to induce different HSP-27 levels among the four groups (Figure 7C), a marked up-regulation of postischemic HSP-27 level was observed in WT and HIF-1α^−/−^ kidneys that were treated with UO plus IR procedures. However, in HIF-2α^−/−^ and DKO kidneys, UO led to much blunted up-regulation of postischemic HSP-27 level (Figure 7D).

### UO led to much better postischemic recovery of renal blood flow, which was totally negated by HIF-2α knockout

**Figure 8:**
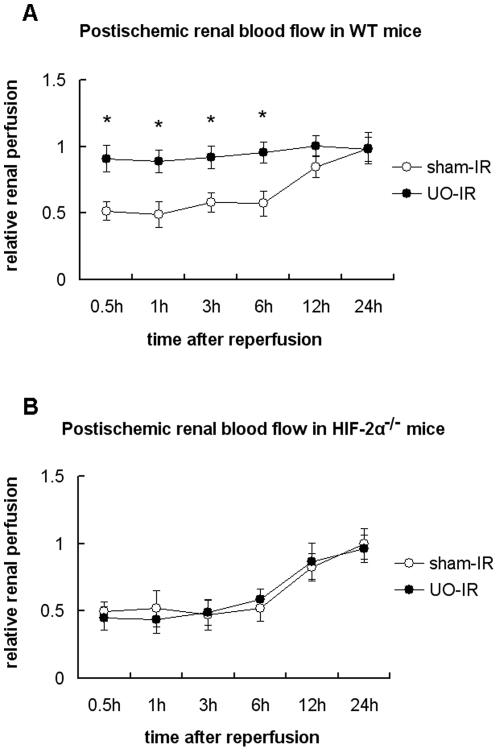
Effect of UO on postischemic recovery of intra-renal microvascular blood flow in WT and HIF-2α^−/−^ mice. WT (A) or HIF-2α^−/−^ mice (B) were subjected to either non-UO sham surgery or left UO, before which baseline renal blood flow was obtained by monitoring the microvascular blood flow in the right kidney. 2 days after release of obstruction, the left kidney was subjected to 25 min of ischemia immediately after right nephrectomy was performed. After the initiation of reperfusion, the blood flow in the left kidney was measured at the indicated time points, and the mean value versus the baseline value was defined as relative renal perfusion. Graph showed data acquired from 4–6 independent experiments for each mouse strain at each time point. *, p<0.05 between UO-IR and sham-IR groups.

To assess the possibility that UO and HIF-2α may act to protect the vascular function and facilitate renal microcirculation following ischemia, renal blood flow in the outer medulla was measured in WT and HIF-2α^−/−^ mice after the initiation of reperfusion. Baseline microvascular flow, which was obtained from the right kidneys prior to UO or sham operations, was comparable between WT and HIF-2α^−/−^ mice. As shown in Figure 8A, after the initiation of reperfusion, renal blood flow didn't recover to the baseline level until 12 hours after reperfusion. But in those with UO preconditioning the blood flow recovered so fast that we called it “an immediate recovery”. However, the “immediate recovery” can only be seen in WT mice. No differences could be observed between UO and sham groups in HIF-2α knockout mice (Figure 8B). These data suggested that HIF-2α was not a regulator of basal renal hemodynamics, but preconditional activation of HIF-2α helped the endothelium to face the acute ischemic challenge and greatly improved the reperfusion efficiency.

## Discussion

Since ischemic acute renal failure continues to be associated with a very high mortality rate in humans, it is important to understand the endogenous processes the kidney uses to protect itself. Several studies provided evidences for a critical role of HIF-1 in the protective effects of ischemic preconditioning in multiple organs [32]–[34]. It was also reported [5], [35] that preconditional pharmacological or genetical activation of HIFs before pathological insult activated a self-defense mechanism and ameliorated ischemic acute renal failure. Park et al. reported [3] that prior transient UO led to markedly alleviated injury in subsequent renal I/R, but the authors did not correlate this phenomenon with the activation of HIFs. It was known that UO was associated with a reduction in renal blood flow and resulted in renal tubular hypoxia [36], [37], and it was also reported [16], [38] that UO led to activation of HIFs. So we had reasons to speculate that the renoprotective effects conferred by UO might be associated with activation of HIFs. Since the transcriptional response to hypoxia was primarily mediated by two hypoxia-inducible factors, HIF-1 and HIF-2, we set out to explore the role of the two HIFs in the renoprotective effects of UO.

We first determined whether transient ligation of the ureter could cause renal hypoxia and accumulation of HIF-α protein in kidney tissues. Previous report [16] has suggested that UO was associated with a reduction in renal tubular oxygen tensions 24 hours after ligation. In that study tissue hypoxia was detected using pimonidazole hydrochloride, which formed protein adducts only in cells that experienced an oxygen level of approximately 1% O_2_, or lower. Here we used a quantitative method, which was based on the principle of oxygen quenching of fluorescence, to reveal the influence of 24 h UO upon the renal tissue oxygen level. The results demonstrate that 24 h of UO leads to a mild hypoxic state in the ipsilateral kidney, and thus transient UO and the following recanalization put the kidney through a hypoxia and re-oxygenation cycle, which makes UO an atypical kind of hypoxic preconditioning.

Although previous study [16] has demonstrated stabilization of HIF-1α and HIF-2α protein in UO kidneys, a kind of continuous UO, which lasted for 8 days, rather than transient UO, was employed in that study. Our data revealed that transient UO, which lasted for only 24 hours, triggered a robust and long-lasting HIF-2α (rather than HIF-1α) accumulation, which continued for over a week and peaked on day 2 after release of obstruction. Previous sublethal hypoxia induces tolerance to subsequent hypoxic/ischemic insults in a process known as ischemic preconditioning or hypoxic preconditioning, in which HIF-1 has been well confirmed as a key transcription protein [32], [39]. HIF-2 has seldom been reported to engage as a participant in hypoxic preconditioning. Our results demonstrate that prior transient UO, as an atypical kind of hypoxic preconditioning, also features activation of an atypical molecular mediator.

To gain further insight into the role of HIFs in UO-related renal protection and to establish whether this relation is causal, we used a well established model of unilateral renal I/R in mice with conditional knockout of HIF-α isoform(s). As was reported, prior transient UO resulted in profound protection against ischemic injury after release of obstruction in WT mice. Inactivation of HIF-2α, rather than HIF-1α, greatly neutralized the renoprotective effects of UO, as indicated by higher creatinine level, severer histological damage and leukocyte infiltration, much more apoptotic cells, as compared with those in WT and HIF-1α^−/−^ mice. HIF-1α/2α double-knockout mice manifested quite similar degree of injury to HIF-2α^−/−^ mice, showing that there wasn't a compensatory effect between HIF-1α and HIF-2α in this setting.

But why is HIF-2α, rather than HIF-1α up-regulated and playing a key role in this setting? The tPO_2_ measurements have shown that UO decreases the renal tissue oxygen level from approximately 30 mmHg to 20 mmHg. So, unlike ischemic preconditioning, which involves total cessation of renal blood flow, UO is a kind of “long-lasting but mild” hypoxic preconditioning. It has been reported [40] that HIF-2α stabilization can be detected in well-vascularized regions and at higher O_2_ tensions (5%, mild hypoxia) than HIF-1α (below 1%, extreme hypoxia). The temporal patterns of the two HIF-α subunit accumulation are also different. HIF-1α is stabilized acutely, whereas HIF-2α protein gradually accumulates and remains stabilized over longer periods of hypoxia, governing prolonged hypoxic gene activation. While HIF-1α and HIF-2α share significant sequence homology, have similar domain architecture and undergo the same proteolytic regulation [41], this study in the context of UO-mediated renoprotection again demonstrates significant differences in the biological characteristics of these two isoforms.

Our results also demonstrated that without UO preconditioning, HIF-1α or 2α knockout didn't lead to altered ischemic renal damage. This was inconsistent with previous observations which demonstrated that a genetic reduction in HIF-1α or HIF-2α led to severer injury [42], [43]. This may be explained by the different gene targeting techniques that were employed. In the previous reports, a systematic knockdown of target genes by standard gene targeting in ES cells led to heterozygous deficiency for HIF-1α or HIF-2α throughout ontogeny, which may lead to developmental defect, especially in vasculature. However, in this study the gene inactivation was induced by pIpC injection in adult animals, and the extent of target gene deletion by Mx-Cre was approximately 50∼60% in the kidney, which comprised a relatively small proportion of interferon-responsive cells [44]. So, although this technique led to homozygous deficiency for target genes in interferon-responsive cells, there were still quite a proportion of renal cells with intact target genes.

HSP-27 functions as an anti-apoptotic molecule and prevents cell death by a wide variety of agents that cause apoptosis [45]. HSP-27 also reduces the inflammatory responses by decreasing production of cytokines and adhesion molecules, reducing leukocyte-endothelial interactions, and mitigating congestion in the outer medulla [3]. Selective renal overexpression of human HSP-27 reduces renal IRI [46]. So, HSP-27 plays a role in the protection against IRI [47], [48]. However, expression of HSP-27 is only transiently induced in response to the stress events, after which expression levels fall drastically, thus allowing only for overexpression when its cytoprotective properties are required. Park et al. reported that UO induced an increase of HSP-27 expression [3]. But why HSP-27 was up-regulated after UO has not been clarified. HSP-27 was reported to be regulated by hypoxic signaling through HIF-1 activation in the retina and to protect the retina from ischemic injury [49]. Our results are significant, not only because we reconfirmed the relation between HIF and HSP-27 expression in another organ, but also because it was the first time HIF-2 activation was demonstrated to be also associated with HSP-27 up-regulation. Although HSP-27 may take a big part in UO-mediated renoprotection, it is unlikely that HSP-27 alone is responsible for all the protective effects. Further studies should clarify whether HIF-regulated hypoxia responsive genes besides HSP-27 are increased in the kidney in response to acute urinary tract obstruction, and whether they are vital to the prevention of renal IRI.

As was reported, HIF-2α was not expressed in renal tubular cells, but in peritubular endothelial cells and fibroblasts [13], [14]. Then how does HIF-2α protect against renal tubular damage?

Although the renal tubular epithelial cell injury that occurs during an ischemic event undoubtedly plays a key role in ischemic renal injury, there is growing evidence that renal vascular endothelial injury and dysfunction are even more important factors in initiating and extending renal tubular epithelial injury [50], [51]. Lack of adequate renal cortical-medullary reperfusion may be more deleterious than the classical “reperfusion injury” secondary to oxygen and nitrogenous free radical formation [52], [53]. Medullary endothelial cell injury and dysfunction may also contribute to the inflammatory response, because endothelial cells in the medullary region, but not the cortex, express surface markers important in lymphocyte activation [54].

Measurement of blood flow changes in the microcirculation using LDF has well proved to be a reliable method for the assessment of endothelial function [55], [56]. So, to explore the possible influence of UO upon postischemic renal endothelial function and the role of HIF-2α in this setting as well, we used LDF to evaluate the renal microcirculatory recovery after I/R insult. In WT mice with UO preconditioning, blood flow in renal outer medulla approached the pre-ischemic level much more rapidly than those without UO pretreatment. However, inactivation of HIF-2α totally negated the effects of UO preconditioning, indicating that activation of HIF-2α by UO reduced renal IR injury via preservation of vital vascular functions and medullar blood flow.

Based on initial reports showing high levels of expression of HIF-2α mRNA in endothelial cells and some highly vascularized tissues, HIF-2α was also referred to as endothelial PAS domain protein 1 (EPAS1) [57]. HIF-2α is actually very important for hypoxic adaptation of the vasculature, which is supported by the observations that HIF-2α has a stronger transactivation activity than HIF-1α on the promotor of vascular endothelial growth factor (VEGF) [58], [59], and overexpression of HIF-2α, but not HIF-1α, was found to enhance expression of the endothelial tyrosine kinase receptor Tie2 [57], [60]. We have correlated HIF-2α activation with up-regulated HSP-27. In fact, an increase in the HSP-27 levels in infected endothelial cells has been demonstrated to correlate well with their resistance to apoptosis under reoxygenation [61].

Previous report [43] using systematic but heterozygous HIF-2α knockdown mice has demonstrated that in the setting of renal IR, there was a specific role of HIF-2α in endothelial cells, but not in inflammatory cells. The authors indicated that HIF-2α knockdown mice were more susceptible to renal IRI, because of peritubular capillary loss and decreased expression of antioxidative stress genes in endothelial cells. In our study, since interferon expression can be induced in endothelial cells[62], and thus floxed target genes can be inactivated homozygously in renal endothelial cells in adults by the Mx-Cre/loxP recombination system [63] without damaging vascular development, these results give further evidence of the crucial role of HIF-2α in the protection against endothelial dysfunction in acute ischemic renal injury.

These results have significant research implications. Since 24 h of UO leads to HIF-2α stabilization without the induction of HIF-1α, UO can be widely employed in the research work concerning the role of HIF-2α in various renal diseases besides ischemic acute renal failure, for example, nephrotoxic acute kidney injury, radiocontrast nephropathy, and acute glomerulonephritis. Our findings may also be of importance to clinical practice. Although UO is not a feasible way to reduce clinical renal IRI, the strong protective effects of UO can be obtained by pharmaceutical activation of HIF-2α. And since the two HIFs can be stabilized under different conditions and mediate different adaptive responses to hypoxia, it might be a viable therapeutic option that exogenous influences could be developed to mimic both processes and create a synergistic effect in attenuating renal IRI.
